# Synthesis, radiolabeling, and evaluation of 68Ga-labeled aminoquinoxaline derivative as a potent PFKFB3-targeted PET tracer

**DOI:** 10.3389/fchem.2023.1158503

**Published:** 2023-03-22

**Authors:** Feng Chen, Yi Wu, Yixuan Ma, Honghai Yin, Feijing Su, Rui Huang, Xiaoai Wu, Qian Liu

**Affiliations:** ^1^ Suzhou Medical College of Soochow University, Suzhou, Jiangsu, China; ^2^ Department of Pediatric Surgery, The First Affiliated Hospital of Gannan Medical University, Ganzhou, Jiangxi, China; ^3^ Key Laboratory of Prevention and Treatment of Cardiovascular and Cerebrovascular Diseases, Ministry of Education, Gannan Medical University, Ganzhou, China; ^4^ Jiangxi Provincial Clinical Research Center for Vascular Anomalies, The First Affiliated Hospital of Gannan Medical University, Ganzhou, Jiangxi, China; ^5^ Department of Nuclear Medicine, Laboratory of Clinical Nuclear Medicine, National Clinical Research Center for Geriatrics, West China Hospital, Sichuan University, Chengdu, China; ^6^ Core Facilities of West China Hospital, Sichuan University, Sichuan, China; ^7^ Department of Neurology, Sichuan Academy of Medical Science and Sichuan Provincial People’s Hospital, Chengdu, China; ^8^ Integrated Chinese and Western Medicine Institute for Children Health & Drug Innovation, Jiangxi University of Chinese Medicine, Nanchang, Jiangxi, China; ^9^ Jiangxi Key Laboratory of TCM for Prevention and Treatment on Hemangioma, Nanchang, Jiangxi, China

**Keywords:** PFKFB3, radiolabeled compounds, inhibitors, PET tracers, PET

## Abstract

Glycolysis, as a multi-step oxidation process, plays important roles in the energy supply for living cells, including malignant tumor cells. Recent studies have revealed that 6-phosphofructo-2-kinase/fructose-2,6-biphosphatase 3 (named PFKFB3), a bifunctional enzyme in glycolysis, is upregulated in a variety of malignant solid tumors and has been regarded as a potential biomarker for the diagnosis and treatment of tumor patients. Based on the structure of selective PFKFB3 inhibitors, we designed and synthesized a radio-metal radiolabeled small molecule, ^68^Ga-5, which also showed potent selectivity in enzymatic and biochemical tests (with an IC_50_ value of 12.5 nM). According to further *in vitro* and *in vivo* evaluations, ^68^Ga-5 showed promising properties as a PET ligand, and selective accumulation in PFKFB3-positive tumors was observed in PET images (with max SUV values of 0.60). Our results indicated that radio-metal radiolabeled aminoquinoxaline derivative, as represented by ^68^Ga-5, held the potential to be developed as selective PFKFB3-targeted PET tracers, and further investigation and optimization would also be required for this scaffold.

## 1 Introduction

As the most important multi-step oxidation process to provide energy from glucose, glycolysis is controlled by a series of bifunctional enzymes and enables the conversion of glucose to pyruvic acid with the release of two molecules of ATP (Vander Heiden et al., 2009; Koppenol et al., 2011; Hay, 2016). The primary rate-limiting step in glycolysis, known as the transformation of F6P to fructose-1,6-bisphosphate (F1-6BP), is catalyzed by PFK-1 and determines the glycolytic flux. However, the activity of PFK-1 was activated by F2-6BP, a product of F1-6BP catalyzed by 6-phosphofructo-2-kinase/fructose-2,6-biphosphatase (named PFKFB). F2-6BP can overturn the inhibition of PFK-1 by ATP as well as other endogenous substrates and enhance the affinity of F6P to PFK-1, allowing increased biosynthesis of F1-6BP and glucose metabolism (Okar et al., 2001; Atsumi et al., 2002; Okar et al., 2004; Cross et al., 2014; Fan et al., 2014; Bousseau et al., 2018; Wang et al., 2020). Therefore, PFKFB plays important roles in cell glucose metabolism.

**FIGURE 1:**
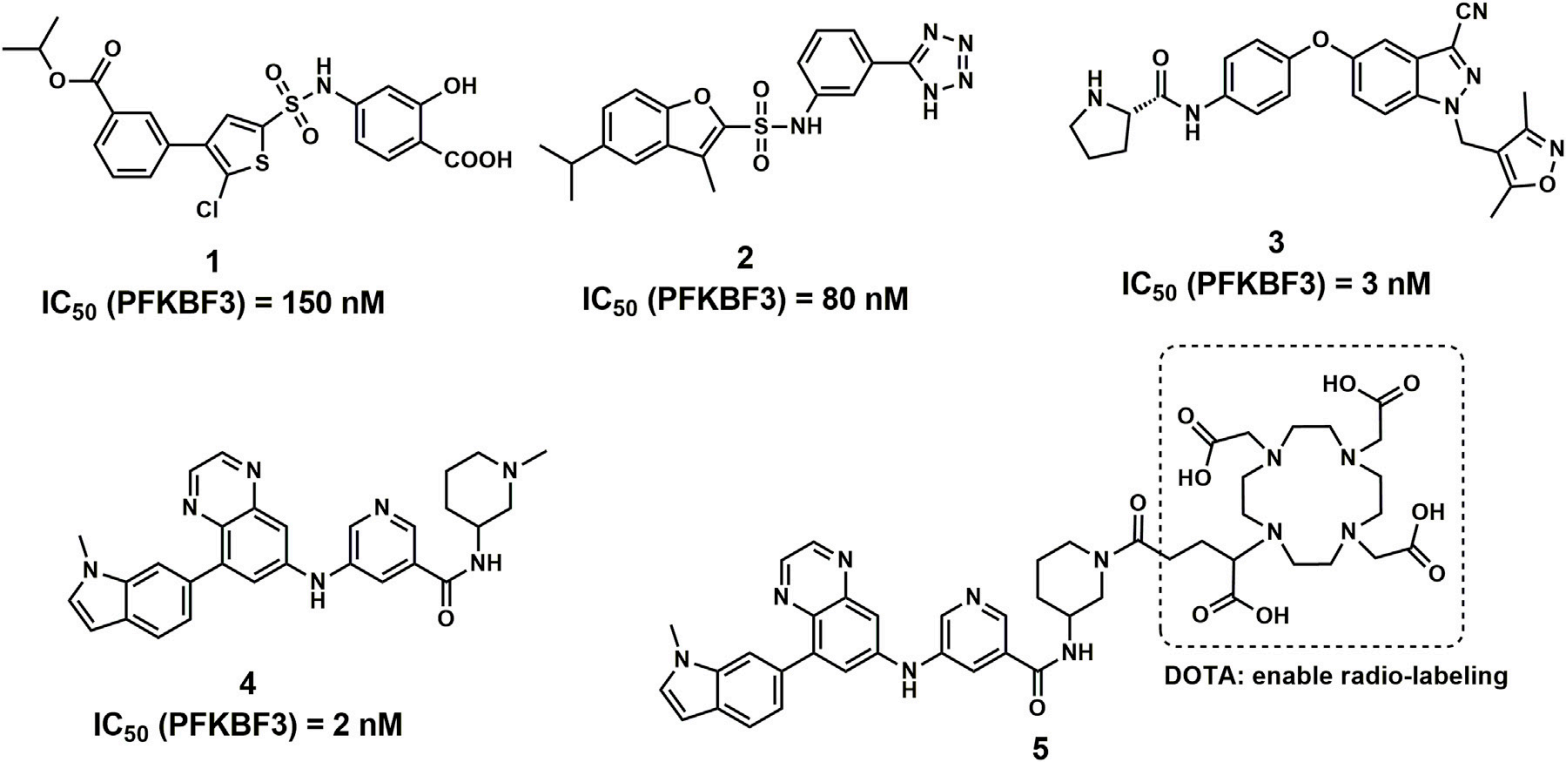
Structures of the representative PFKFB3 inhibitors (compounds 1–4) and compound 5.

PFKFB3 is one of the major isozymes of PFKFB1-4 and is frequently overexpressed in a variety of human solid tumors, including breast, lung, gastric, pancreatic, ovarian and colon cancers (Chesney et al., 1999; Atsumi et al., 2002; Calvo et al., 2006). In addition, PFKFB3 showed the most potent activity compared with the other 3 isozymes, making it a driving force of cancer oxygen-independent glycolysis (Chesney et al., 1999; Fukasawa et al., 2004). Therefore, PFKFB3 has attracted much attention and is regarded as a promising target for cancer therapy, and many novel compounds have been reported with potent PFKFB3 inhibitory activities (Figure 1) (Boyd et al., 2015; Boutard et al., 2019; Wang et al., 2020).

Radio-metal labeled compounds provide an attractive strategy for the diagnosis and treatment of tumors. With the “therapeutic pairs” of diagnostic and therapeutic nuclides labeled on the same site (usually DOTA and NOTA) of one highly sensitive molecule, the detection, diagnosis, and treatment of tumors *via* specific targets by one single molecule can be achieved. (Drude et al., 2017). For example, ^68^Ga-FAPI, ^68^Ga-PSMA, and ^68^Ga-DOTATATE were used for tumor diagnosis by PET, while ^177^Lu-FAPI, ^177^Lu-PSMA, and ^177^Lu-DOTATATE have also been evaluated in tumor treatment in the clinic (Drude et al., 2017; Lindner et al., 2019; Sun et al., 2020; Strosberg et al., 2021).

With highly potent and selective activity against PFKFB3, the scaffold of aminoquinoxaline derivatives (compound 4) attracted our attention (Boutard et al., 2019). With the goal to develop novel PFKFB3 PET tracers for tumor diagnosis, we performed the structural modification to the aminoquinoxaline scaffold and radiolabeled with ^68^Ga for *in vitro* and *in vivo* evaluations. With a similar structure of glucose, ^18^F-fluorodeoxyglucose (^18^F-FDG) is able to visualize the abnormal glucose metabolism and hence has been widely used for the diagnosis of tumors (Jadvar et al., 2009; Zhu et al., 2022). However, the accumulation of ^18^F-FDG mainly revels the activity of glucose transporters (GLUT) in tumor cells and tissues, the biomarker for tumor glycolysis may play more important role in tumor diagnosis, such as PFKFB3 (Ganapathy-Kanniappan and Geschwind, 2013; Meziou et al., 2020). Although ^68^Ga-FAPI is a rising star in tumor diagnosis as a broad-spectrum PET tracers as ^18^F-FDG, the false-positive uptakes were observed and reported in bone, breast and other benign lesions (Gündoğan et al., 2021; Song et al., 2021; Kessler et al., 2022). Therefore, the development of PFKFB3 targeted PET tracers may of great significance for the complement of ^18^F-FDG PET.

In this study, a potent ^68^Ga radio-labeled aminoquinoxaline derivative (^68^Ga-5) was successfully produced and evaluated. Our results indicate that aminoquinoxaline derivatives have the potential to be developed as a “theranostic” probe for tumor diagnosis and treatment, but further comprehensive studies are needed to fully investigate the SAR of this scaffold with PFKFB3.

## 2 Materials and methods

### 2.1 Molecular docking studies

Molecular docking study was performed to predict and evaluate the binding model of compound 5 with PFKBF3. All of the calculations were based on Discovery Studio 3.1 (Accelrys Inc., San Diego, CA, United States). The protein structure was prepared with the crystal structure of PFKBF3 (PDB ID: 6IBX). The binding site was configured as a sphere with the residues that stay within 10 Å diameter from its original ligand (compound 4), which is large enough to incorporate the ATP binding pocket of the PFKBF3 interface. The GOLD program was used to perform the docking calculations.

### 2.2 General information for chemistry

All regents and solvents were purchased from commercial suppliers and used in this study without further handling unless indicated. Thin-layer chromatography and flash column chromatography were used to monitor and purify organic reactions. Nuclear magnetic resonance (NMR) for ^1^H and ^13^C NMR spectra was carried out on a Bruker spectrometer (Bruker AV-400, 400 MHz, United States) in the Analysis and Testing Center of Sichuan University. The coupling constants are calculated and presented as J with Hz (hertz), and singlet, doublet, triplet, quartet and multiple are displayed as s, d, t, q, and m, respectively. The mass spectra were obtained on an Agilent 6125 LC/MSD system. As a positive control, compound 4 was synthesized with an identical procedure from published literature, which was also modified for the synthesis of compound 5 (Boutard et al., 2019). All the synthesis procedures and characterization data can be found in the Supplementary Materials.

### 2.3 Biochemical activity and cellular inhibition assay

All regents used in this section were purchased from Sigma, and the Human Recombinant PFKFB3 Protein (His and GST Tag) was purchased from SinoBiological. The ADP-Glo^™^ Kinase Assay kit was also obtained from Promega.

The PFKFB3 enzymatic activity assay followed an identical procedure used in previously published literature, along with the same regents and materials (Boutard et al., 2019).

For the cellular inhibition assay, the protocol used in this section was also from the same literature as the PFKFB3 enzymatic activity assay, with minor modifications (Boutard et al., 2019). Briefly, glucose-starved HTC116 cells were pretreated with diluted compounds at different concentrations and restimulated with 25 mM glucose for 4 h to trigger glycolytic flux. Cells were then lysed by NaOH (0.1 mM) under 85°C for 10 min and transferred to a HEPES solution (20 mM) neutralized by acetic acid (1 M) to pH 7.5–8.0. The lysates (equal amounts, normalized to protein level) were reacted in a mixture of α-glycerophosphate dehydrogenase (0.17 U), aldolase (0.083 U), pyrophosphate-dependent fructose-6-phosphate kinase from potato (0.17 U) and triosephosphate isomerase (0.83 U) in Tris (pH 8.0), glucose-6-phosphate (17 mM), fructose-6-phosphate (2.5 mM) and magnesium chloride (5.5 mM) in a total volume of 100 µL. The reaction was induced by the addition of NADH (0.14 mM) under 30°C, and the absorbance at 340 nM was measured 30 min post addition. All absorbances for reaction mixtures for test compounds with different concentrations were recorded and used for IC_50_ calculations in GraphPad Prism 5.0 software.

### 2.4 Radiolabeling and characterization

The ^68^Ga-5 was prepared following a general procedure for ^68^Ga radiolabeling (De Silva et al., 2018). Briefly, the ^68^Ge/^68^Ga generator (Eckert and Ziegler) was eluted with 10 mL of 0.1 N HCl solution (99.99%, trace metals basis grade), the first fraction (2.5 mL) showed low activity and was discarded, and the next fraction (2.0 mL) with approximately 85% radioactivity was collected for radiolabeling. In a glass vial containing 50 µg of compound 5 (lyophilized powder) was added 40 µL of 1 N sodium acetate buffer and 400 µL of generator elution. The pH value of the final reaction mixture was about 3.5–4.0. The reaction was performed under 90°C for 20 min with a block heater. The reaction mixture was then passed through a Waters C-18 September-Pak light cartridge and rinsed with 20 mL of distilled water, and the radioactivity trapped in the C-18 cartridge was then washed with 400 µL (2 × 200) of ethanol. The ^68^Ga-5 in ethanol was then formulated in 5 mL of saline for further evaluations. The radio-chemical purity (RCY) of the final product was assessed by an Agilent HPLC 1100 system equipped with a Phenomenex C-18 Luna column (5 μm, 10 * 250 mm), and an FC3200 gamma detector (BioScan) was used to collect the gamma signal. The mobile phase of A (MeCN) and B (0.1% TFA in water) was used in the analysis based on a gradient method (from 0–1 min isocratic 30% A and 70% B; from 1–10 min gradient A 30%–100%, B 70%–0%; from 10–11 min gradient A 100%–30%, B 0%–70%; from 11–15 min isocratic 30% A and 70% B), with a flowrate set as 1 mL/min.

### 2.5 *In vitro* physicochemical property evaluations

The stability of ^68^Ga-5 was determined in PBS saline, 80% EtOH solution and rat serum. Briefly, 600 µL of the final ^68^Ga-5 solution was added to 2 mL of PBS saline, 80% EtOH solution and rat serum with each 200 µL. These solutions were then incubated in a water bath under 37°C for 4 h, and samples (200 µL) from these solutions were obtained at 1 h, 2 h, 3 h, and 4 h after incubation. Samples from PBS saline and 80% EtOH solution were loaded into the radio-HPLC for RCP analysis as described in the radiolabeling and characterization section. For samples from rat serum, 200 µL of MeCN was added to denature the plasma protein, and then the mixture was centrifuged at 5,000 RPM to precipitate the denatured protein. The supernatants of the centrifuged solution were then applied to HPLC for analysis. The RCP of all samples was calculated based on the area under the curves generated by the gamma detector.

The lipophilicity of ^68^Ga-5 was measured and presented as the partition coefficient at pH 7.4 (Log D_7.4_) as described previously (Li et al., 2019). Briefly, log D_7.4_ was calculated based on the ratio of radioactivity concentrations (radio-counts in gamma counter) in 1-octanol and in PBS saline (pH 7.4). A series of solutions were prepared and tested until a constant value of log D_7.4_ was obtained.

### 2.6 Cellular uptake studies

H1975, HCC827, MKN45 and NUGC3 cells (obtained from ATCC) were investigated in this section. Briefly, cells were cultured, transferred, and plated in 60-mm culture dishes (about 5 × 10^6^ cells per well) overnight for attachment before use. For cellular uptake, approximately 370 KBq of ^68^Ga-5 (aqueous solution with 8% ethanol, approximately 50 µL–100 µL) was added into the cells and incubated under 37°C for 1 h. At each time point, i.e., 15, 30, 45, 60 min, the medium in the well was transferred, and then the well was washed with 2 mL of cold PBS. The medium and the PBS were combined and counted for radioactivity in a gamma counter (Perkin Elmer 2,470 wizard, United States). The cells were then dissolved in NaOH (1 N), and the lysate was also counted in the gamma counter. For blocking studies, all cells were pretreated with 1 µM unlabeled compound 5 1 h before the addition of ^68^Ga-5, and the subsequent procedure for medium and cells was the same as described above. The uptake ratio was then calculated by the following formula: Uptake = (counts in cells)/(counts in medium and PBS) * 100%.

### 2.7 Biodistribution studies

All protocols and procedures in this study were approved by the animal care and use committee of Sichuan University. To evaluate the *in vivo* pharmacokinetic properties of ^68^Ga-5, a tissue biodistribution study was performed in normal Kunming mice. Subjects were grouped by time points, i.e., 5, 15, 30, 60, 90, and 120 min (*n* = 5), and were administered 30 µCi of ^68^Ga-5 by tail vein injection under anesthesia. At the designated time points, subjects were sacrificed. The tissues of interest were collected, weighed, and counted in a gamma counter (Perkin Elmer 2,470 wizard, United States) for radioactivity. The tissue distribution was then calculated and presented as the percentage of the injected radioactivity dose per gram of tissue (%ID/g, decay-corrected).

### 2.8 Animal models and micro-PET imaging

BALB/c nude mice (18–20 g, 4–5 weeks) were subcutaneously injected with tumor cells suspended in 100 µL of PBS (5 × 10^6^ cells) in the left or right axilla. The tumor models were ready to use when the tumor volume reached 500 mm^3^ about 10–15 days postinoculation. Small animal PET imaging studies were carried out on a micro-PET/CT system (IRIS, Inviscan, France). Briefly, tumor-bearing mice were injected with approximately 3.74 MBq of ^68^Ga-5 or ^18^F-FDG with tail vein administration under anesthesia. For blocking studies, subjects were pre-injected with unlabeled compound 5 (5 mg/kg) *via* the tail vein 1 h before the administration of ^68^Ga-5 and static PET images were obtained at designated time points, i.e., 15, 30, 60, and 90 min, which were reconstructed by a 3D-OSEM algorithm and a Monte Carlo-based accurate model. Regions of interest (ROIs) for major organs were drawn directly from the PET images, and the standardized uptake value (SUV) of all ROIs was also obtained directly from static PET images.

### 2.9 Fluorescence microscopy studies

After PET imaging, fluorescence microscopy studies were then used to evaluate PFKFB3 expression in all tumors included in this study. All regents used in this section were purchased from Sigma. Anti-PFKFB3 antibody and goat anti-rabbit IgG H & L (FITC) were purchased from Abcam. A standard immunofluorescence procedure was performed to handle the tumor sections that were removed from the subjects after small PET imaging.

## 3 Results

### 3.1 Molecular docking studies with the designed compound

**FIGURE 2:**
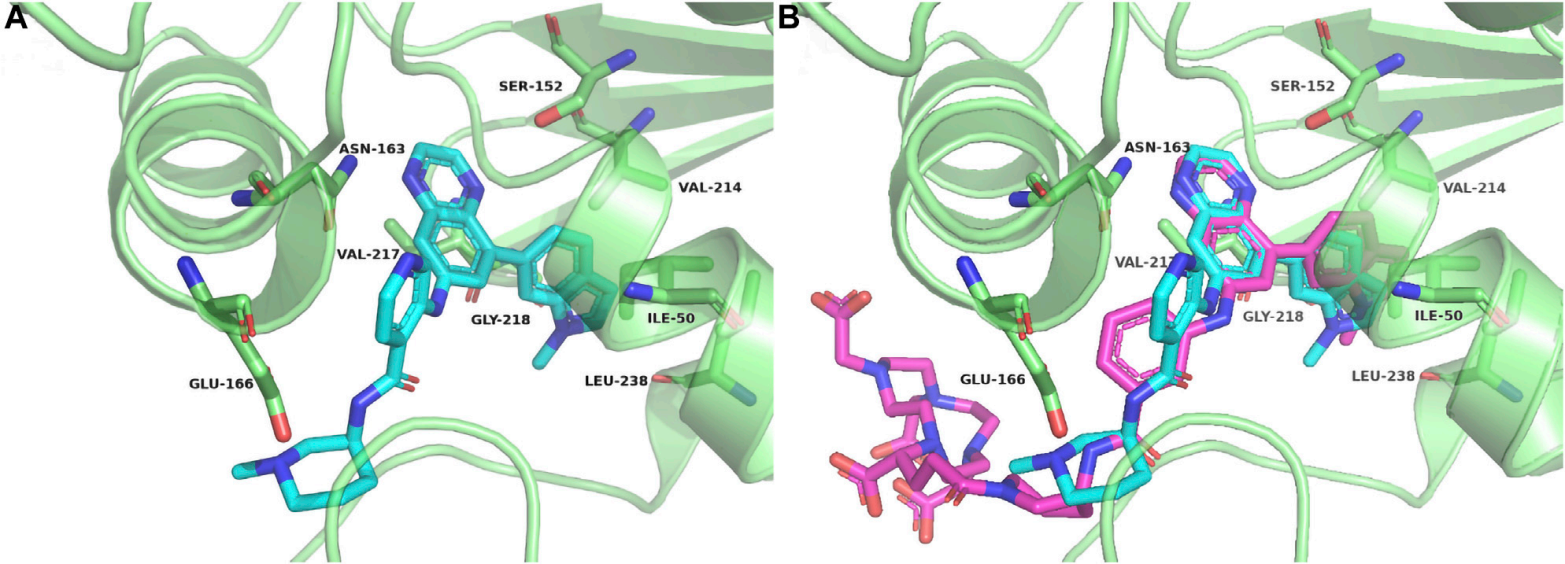
Crystal complex of compound 4 with PFKFB3 (PDB code 6IBX) **(A)** and overlay between compounds 4 and 5 with PFKFB3 **(B)**.

According to the cocrystal structure of compound 4 with PFKFB3, a number of important interactions can be confirmed, such as characteristic hydrogen bonding interactions and hydrophobic contacts, as well as the salt bridge between Glu166 and the ammonium nitrogen atom (Boutard et al., 2019). In addition, we also observed the methyl group at the “left” side of compound 4, which was toward the outer space of the ATP binding site of PFKFB3. Therefore, we have confidence in introducing a large chemical group such as DOTA into the left side of compound 4 to produce the potential theranostic compound (compound 5). Based on the docking studies, compound 5 overlaid well with compound 4, placing the “right” side of the molecule in a similar position and placing all relevant nitrogen atoms in a similar place, suggesting high potential to form interactions with PFKFB3, as shown in Figure 2A. In addition, the DOTA group was introduced *via* the nitrogen atom on the piperazine ring of compound 5 and was placed in the outer space of the binding pocket, suggesting a minimal impact on the inhibitory potency (Figure 2B).

### 3.2 Chemical synthesis of the designed compound

**SCHEME 1:**
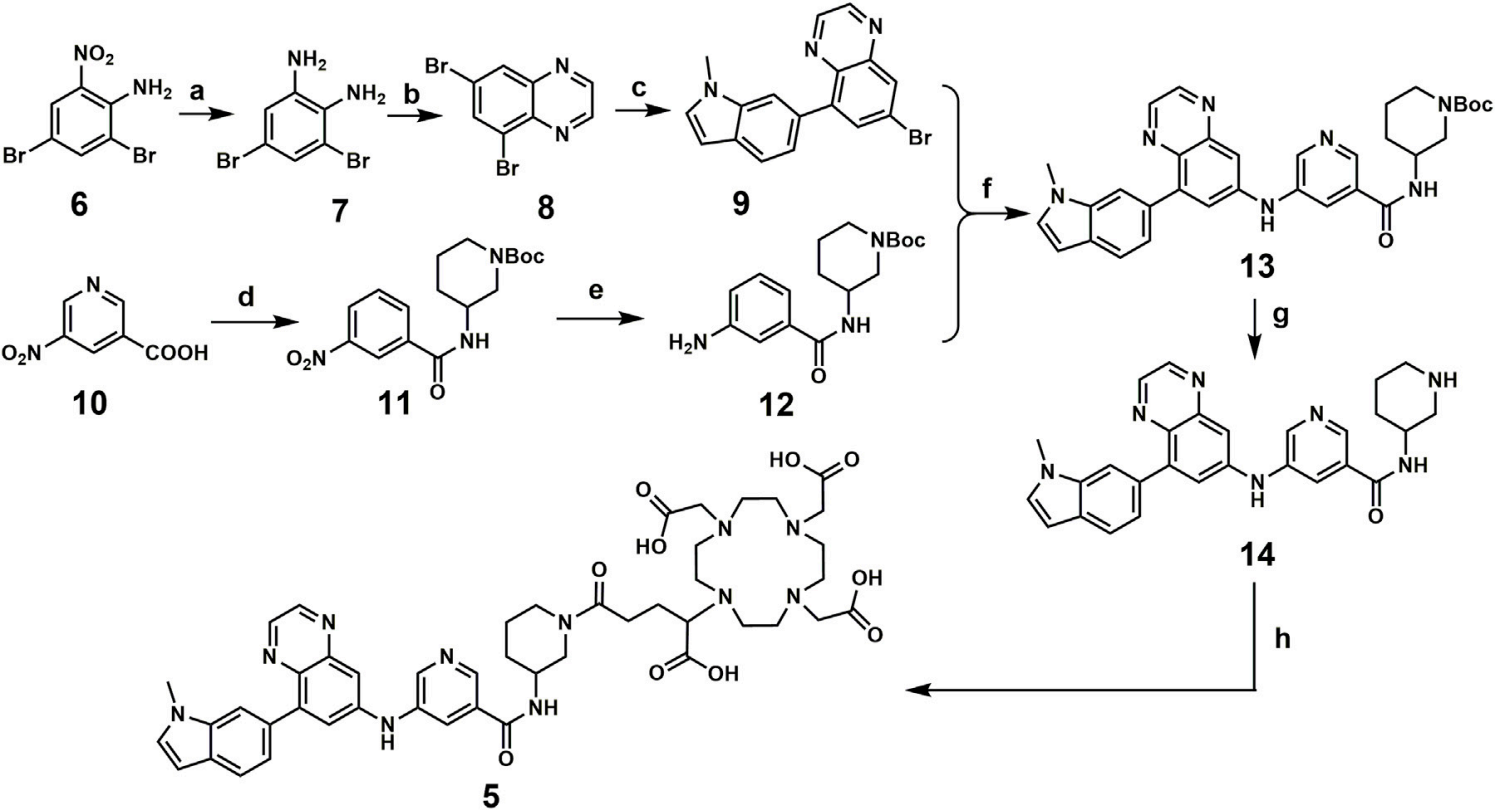
Chemical synthesis route for compound 5.

Compound 5 was synthesized following the published literature, with minor modifications as the synthesis route presented in Scheme 1. First, intermediate 7 was prepared through the reduction of the commercially available nitro precursor (compound 6) and reacted with 1,4-dioxane-2,3-diol to produce the key intermediate 5,7-dibromoquinoxaline (compound 8). Based on the Pd-catalyzed selective coupling reaction, 7-bromo-5-(1-methyl-1H-indol-6-yl) quinoxaline (compound 9) was successfully prepared. Second, tert-butyl 3-(3-nitrobenzamido) piperidine-1-carboxylate (compound 11) was prepared by the amidation reaction of 5-nitronicotinic acid (compound 10) and tert-butyl 3-aminopiperidine-1-carboxylate and was reduced by iron powder to prepare tert-butyl 3-(3-aminobenzamido) piperidine-1-carboxylate (compared 12). Tert-butyl 3-(5-((8-(1-methyl-1H-indol-6-yl) quinoxalin-6-yl) amino) nicotinamido) piperidine-1-carboxylate (compound 13) was successfully prepared *via* the coupling reaction, followed by a deprotecting reaction to yield 5-((8-(1-methyl-1H-indol-6-yl) quinoxalin-6-yl) amino)-N-(piperidin-3-yl) nicotinamide (compound 14). The final product for radiolabeling, 2,2′,2′′-(10-(1-carboxy-4-(3-(5-((8-(1-methyl-1H-indol-6-yl) quinoxalin-6-yl) amino) nicotinamido) piperidin-1-yl)-4-oxobutyl)-1,4,7,10-tetraazacyclododecane-1,4,7-triyl) triacetic acid (compound 5), was produced by an amidation reaction with 2,2′,2′′-(10-(2,6-dioxotetrahydro-2H-pyran-3-yl)-1,4,7,10-tetraazacyclododecane-1,4,7-triacetic acid (DOTA-GA anhydride). In conclusion, the compound was successfully prepared through an 8-step convergent synthesis route with a total yield of 2.51%. In addition, compound 4 was also synthesized as a positive control with an identical procedure published previously.

**Regents and conditions:** a. Iron powder, H_2_O, EtOH, 78°C, 3 h; b. 1,4-dioxane-2,3-diol, EtOH, r.t., overnight; c. Pd (dppf)Cl_2_, DIPEA, 1,4-Dioxane, H_2_O, N_2_, 85°C, 4 h; d. EDCI, HOBT, 4-Methylmorpholine, tert-butyl 3-aminopiperidine-1-carboxylate, r. t, 4 h; e. Iron powder, H_2_O, EtOH, 78°C, 4 h; f. tBuOK, Pd(OAc)_2_, BINAP, toluene, 100°C, overnight; g. TFA, CH_2_Cl_2_, r. t, overnight; h. DOTA-GA anhydride, Et_3_N, DMSO, r. t, overnight.

### 3.3 Biochemical activity and cellular inhibition assay

According to the ADP-Glo^™^-based PFKFB3 activity inhibition assay and a modified procedure for the cellular inhibition assay, compound 4 and compound 5 showed similar enzymatic IC_50_ values for PFKFB3 inhibition and IC_50_ values for inhibition of F2,6BP production in HCT166 cells *in vitro*. As shown in the table, the IC_50_ values for 4 and 5 for PFKFB3 activity inhibition were 6.7 nM ± 2.3 nM and 12.5 nM ± 4.5 nM, respectively. The IC_50_ values for F2,6BP production in HCT116 cells were 2.3 µM ± 0.8 µM and 5.3 µM ± 1.4 µM, respectively. These results indicated that the introduction of a chelating group (DOTA) led to a negligible impact on the inhibitory activity against PFKFB3. In addition, IC_50_ values of 2 nM and 8.37 µM were reported for the enzymatic PFKFB3 inhibition and cellular F2,6BP production assay (Boutard et al., 2019).

### 3.4 Radiolabeling

**FIGURE 3:**
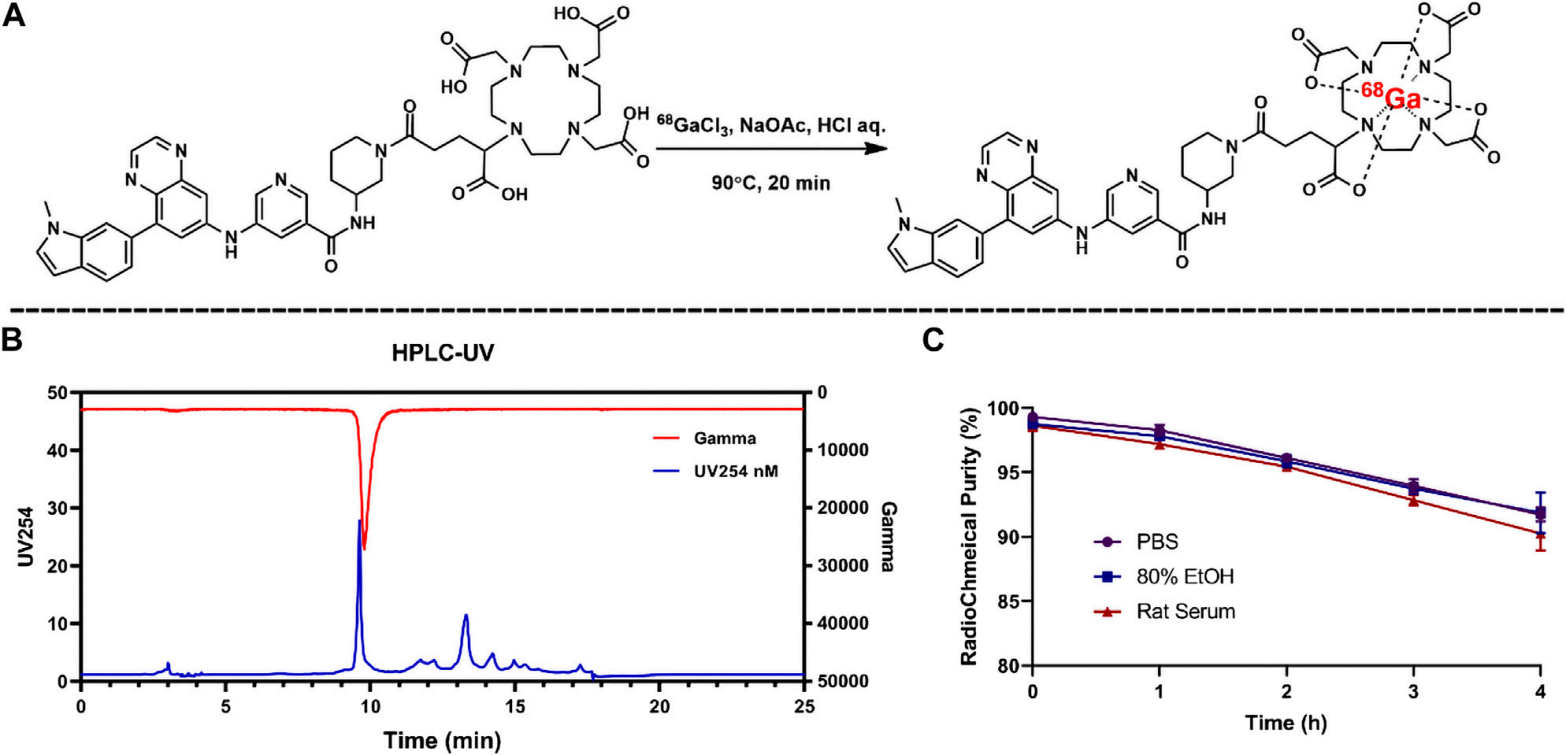
Radiolabeling reaction for ^68^Ga-5 **(A)** and the results for radio-chemical purity **(B)** and *in vitro* stabilities **(C)**.

Radiolabeling of compound 5 was performed manually with high yield under 90, pH 3.5. According to the radio-HPLC profile, the radiochemical purity (RCP) of ^68^Ga-5 was 94.5% + 4.2% (*n* = 5) after an SPE purification procedure. The radiolabeling reaction and the representative HPLC diagram are presented in Figure 3. ^68^Ga-5 showed a retention time of 7.8 min, which corresponds to the UV signal of compound 5.

### 3.5 *In vitro* physicochemical property evaluations

According to the HPLC results for the ^68^Ga-5 solutions of PBS saline, 80% EtOH, and rat serum, ^68^Ga-5 showed strong *in vitro* stability, and over 90% RCP was calculated from the HPLC diagram after 4 h incubation. The l*og D*_*7.4*_ value of ^68^Ga-5 was −1.65 ± 0.28, indicating a much lower lipophilicity than compound 4 (with Log *p* values of 2.22 obtained from ChemBioDraw 14.0 predictions).

### 3.6 Cell uptake studies

**FIGURE 4:**
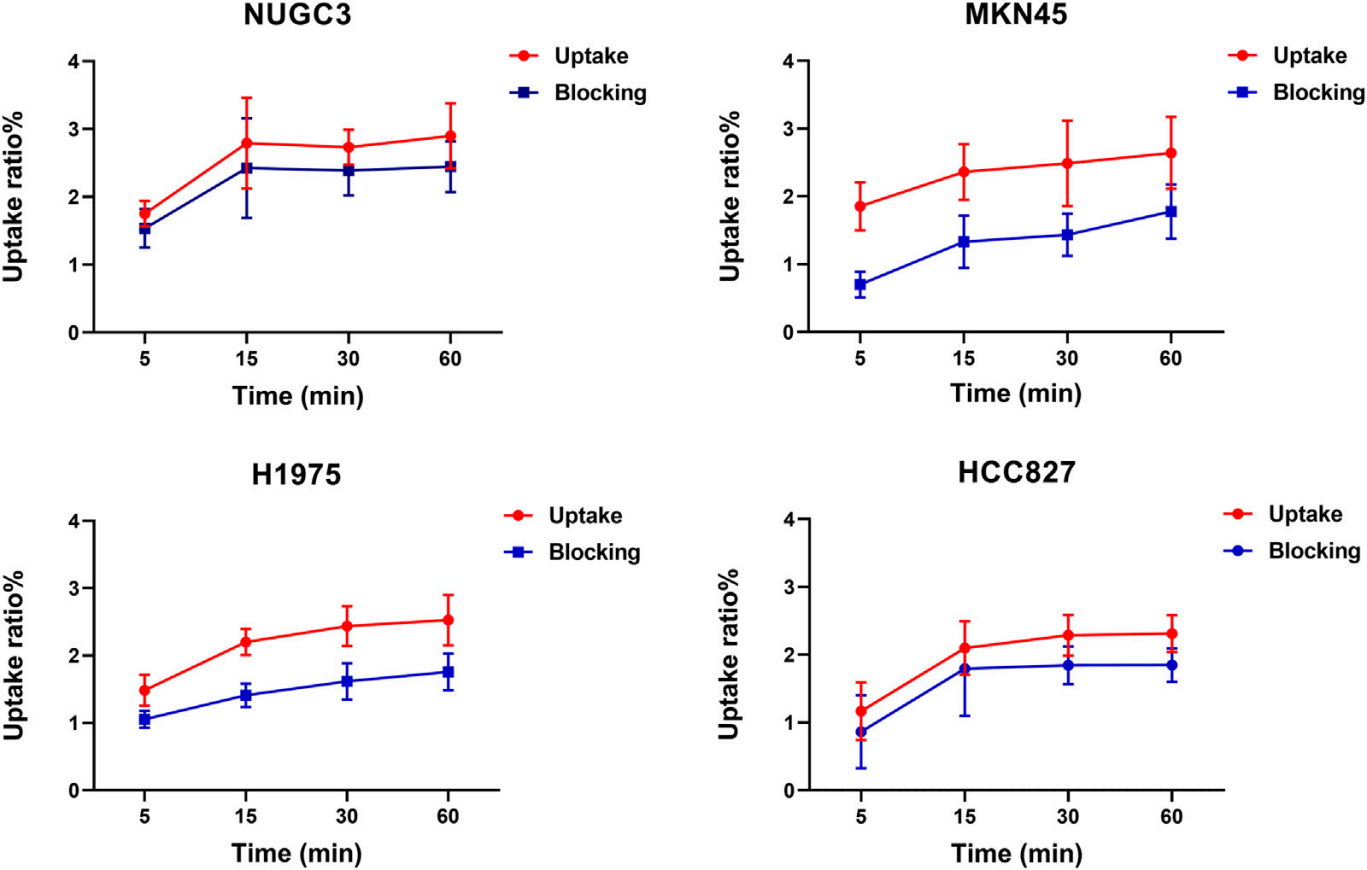
Cell uptake studies of ^68^Ga-5 in selected cell lines.

As shown in Figure 4, ^68^Ga-5 showed preferential accumulation in all tested cells, with uptake ratios of 2.9%, 2.64%, 2.52%, and 2.31% for NUGC3, MKN45, H1975, and HCC827 cells after 1 h of incubation., respectively, In addition, the uptake in these cells can be effectively blocked by the unlabeled precursor (2 µM of compound 4), with uptake ratios decreased to 2.44%, 1.77%, 1.76%, and 1.84%, respectively. NUGC3 showed the highest uptake ratios as well as the largest blocking efficacy (uptake decreased to 84% compared with the unblocked group). As PFKFB3 is widely expressed in almost all types of tumor cells, the uptake ratios and the corresponding blocked uptake ratios indicated that the accumulation of radioactivity in cells is selective and could be mediated by PFKFB3.

### 3.7 Biodistribution studies of ^68^Ga-5 in normal Kunming mice

**FIGURE 5:**
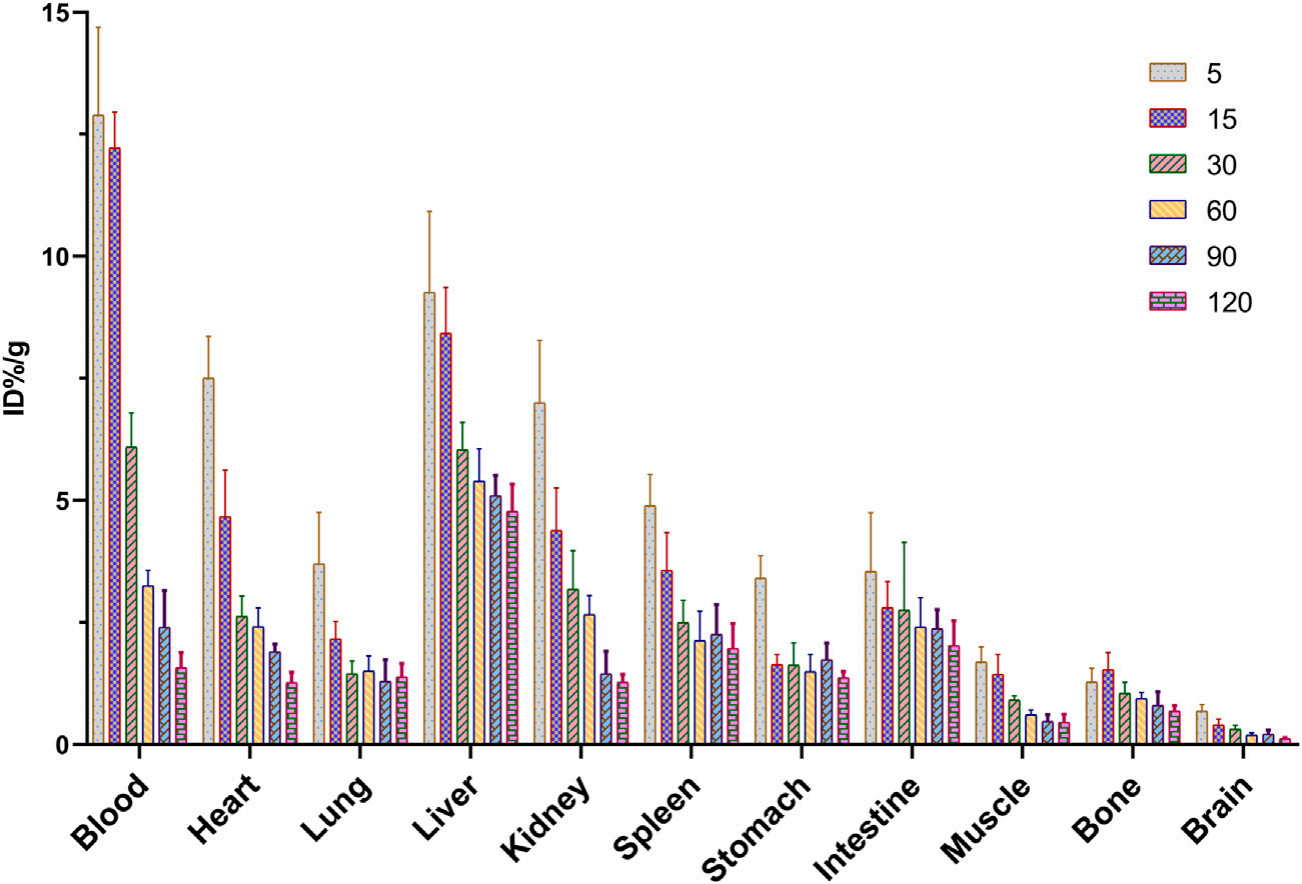
Biodistribution of ^68^Ga-5 in normal Kunming mice.

The tissue biodistribution profile is presented in Figure 5. In normal Kunming mice, ^68^Ga-5 showed moderate pharmacokinetic properties, and the radioactivity in the blood and other major organs slowly decreased post injection. The liver showed the highest initial accumulation of radioactivity with an uptake ratio of 9.26%ID/g at 5 min post-injection (p.i.), and the accumulation decreased to 4.77%ID/g at 120 min p.i., indicating major hepatobiliary excretion. A notable distribution of radioactivity in the kidney was also observed, with accumulation of 7.00%ID/g and 1.27%ID/g at 5 min and 120 min p.i., respectively. The accumulation in the kidney indicated minor urinary excretion of ^68^Ga-5. Interestingly, a higher accumulation in the lung was observed with 3.70%ID/g at 5 min p.i., perhaps due to the large radioactive particles blocked in capillaries of the lung or contamination during organ harvest. Other organs and tissues showed no abnormal accumulation of radioactivity, such as the heart, stomach, muscle, bone and brain, with accumulation data of 1.27, 1.37, 0.45, 0.68, and 0.12%ID/g at 120 min p.i., respectively. Based on the biodistribution profile of ^68^Ga-5, we have the confidence to perform subsequent PET imaging studies.

### 3.8 PET imaging studies

**FIGURE 6:**
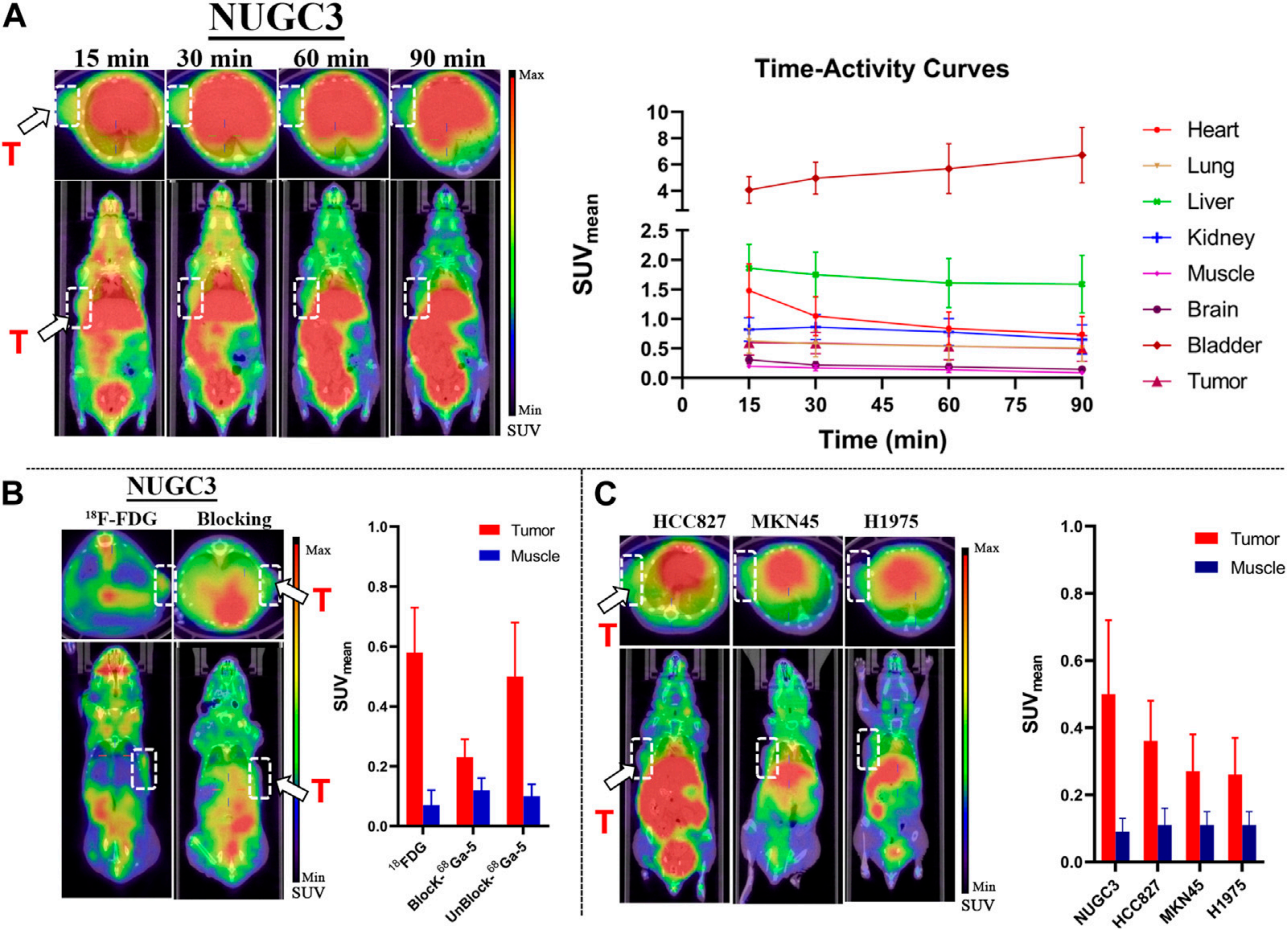
Results for micro-PET imaging studies. **(A)** Representative static PET images of nude mice bearing NUGC3 tumors (left) and time-activity curves generated from static PET images (*n* = 3) (right), stable SUV (0.5–0.7) values in tumor can be observed, with higher SUV values in liver and kidney in all time points. **(B)** Representative static PET images of ^18^F-FDG in the NUGC3 tumor model (90 min) and PET images of the blocking study with unlabeled compound 5 (90 min), 18F-FDG showed higher “Tumor-to-muscle” ratios than ^68^Ga-5 in NUGC3 tumors, and the blocking is significant. **(C)** Representative static PET images of ^68^Ga-5 in mude mice bearing MKN45, H1975 and HCC827 tumors at 90 min p.i. White arrow, white circle and “T” indicate tumor regions.

Representative static PET images at different time points and the corresponding TACs (Time-Activity Curves) are presented in Figure 6. Significant accumulation of radioactivity in the liver, kidney, bladder, and lower digestive tract was observed in all types of tumor mouse models at the end of the scan (Figure 6A–C), which agreed well with the biodistribution profiles. For all types of tumor models, tumor regions were clearly observed at 90 min p.i. As shown in TACs for major organs in NUGC3 models (Figure 6B), the liver showed high initial uptake at 15 min p.i., (with SUV value of 1.86), which decreased gradually and reached 1.59 at 90 min p. i. The bladder displayed the highest SUV values during the scan (with 4.06 at 15 min and 6.72 at 90 min). A fast uptake of ^68^Ga-5 in NUGC3 tumors can be observed and maintained afterwards, with SUV values of 0.60 and 0.50 at 15 min and 90 min p. i., respectively. With the clearance of radioactivity from major organs and muscle, a higher “Tumor-to-muscle” ratio can be obtained. In addition, significant uptake in the lung was also observed with SUV values ranging from 0.51 to 0.65 for all types of tumors included in this study, which agreed with the results of biodistribution studies. However, the intestine and urinary system showed higher uptake which may resulted from the lipophilicity of ^68^Ga-5, as also presented in Figure 6A–C. In blocking studies, the administration of unlabeled compound 5 significantly inhibited the uptake of ^68^Ga-5, with the tumor SUV value decreasing to 0.23 at 90 min in NUGC3 models. In the same subject, the NUGC3 tumor was also clearly visualized by ^18^F-FDG PET with a tumor SUV value of 0.54 at 90 min p.i. 18F-FDG not only showed higher SUV values than ^68^Ga-5 in NUGC3 tumors (0.54 VS. 0.50 @ 90 min p.i.), but also showed higher “Tumor-to-muscle” ratios (8.28 VS. 5.00 @ 90 min p.i.). In addition, ^68^Ga-5 showed much higher background uptake in internal organs such as liver, kidney, spleen, stomach and small intestine, making the quality of tumor imaging is much lower than that of ^18^F-FDG. Tumor uptake was also confirmed in mude mice bearing MKN45, H1975 and HCC827 tumors, with SUV values of 0.36, 0.26 and 0.27 at 90 min p.i (compared with the mean SUV 0.50 for NUGC3 tumors), respectively. The “Tumor-to-muscle” ratios for NUGC3, MKN45, H1975 and HCC827 models at 90 min p.i. are 5.50, 3.27, 2.45, and 2.36, respectively. For 18F-FDG and block studies in NUGC3 tumor models, the “Tumor-to-muscle” ratios are 8.28 and 1.91 (compared with 5.50 for non-blocking group), indicating the tumor angiogenesis is success and the blocking efficacy is significant in NUGC3 tumor models.

### 3.9 Fluorescence microscopy studies

**FIGURE 7:**
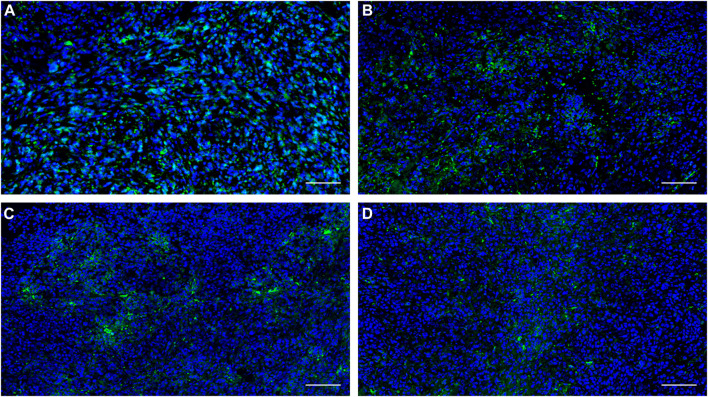
Expression of PFKFB3 in the indicated human tumor tissues was analyzed using immunofluorescence staining. **(A)** NUGC3; **(B)** HCC827; **(C)** MKN45, **(D)** H1975. Scale bar, 100 µm.

In further immunofluorescent staining of tumor tissues, we observed the binding of the antibody to the tumor cells, which confirmed the positive expression of PFKFB3 in all cell lines and cancer tissues used in this study (Figure 7).

## 4 Discussion

As a glycolysis-related enzyme, PFKFB3 is emerging as a potential target for tumor diagnosis and treatment in that it is highly expressed in almost all tumor tissues. In contrast to specific targets that are only expressed in specific types of tumors (such as prostate specific membrane antigen, PSMA), PFKFB3 holds the potential to be regarded as a broad-spectrum target for PET tracer development. ^18^F-FDG and ^68^Ga-FAPIs have been widely used in clinical for the diagnosis of a variety of solid tumors as powerful tools, but the false positive results are also reported which may affect the diagnostic efficacy. To develop highly potent, selective and sensitive PET tracers for the complement of ^18^F-FDG PET and ^68^Ga-FAPI PET is of great importance. Aminoquinoxaline derivatives showed potent activities against PFKFB3, and our interest in this scaffold lies in the convenient conversion of the most potent molecule, compound 4, to a ^68^Ga-labeled PET tracer (^68^Ga-5) using DOTA as a chelator. ^68^Ga-5 was prepared with high radio-chemical yield and radio-chemical purity, and the *in vitro* tests indicated that ^68^Ga-5 had strong stability and moderate lipophilicity. The selective accumulation of ^68^Ga-5 was confirmed by cell uptake studies with NUGC3, HCC827, MKN45 and H1975 cells. Biodistributions of ^68^Ga-5 revealed its moderate pharmacokinetic property, and primary hepatobiliary excretion was observed. In micro-PET imaging studies, the highest SUV values were obtained in NUGC3 tumors (0.5 at 90 min), followed by HCC827 (0.36), MKN45 (0.27) and H1975 (0.26), which agreed with the cell uptake studies. Tumor uptake can be blocked by the pre-injection of unlabeled compound 5 (5 mg/kg), suggesting that tumor uptake is selective. Furthermore, immunofluorescence microscopy images also confirmed the positive expression of PFKFB3 in all types of tumors included in this study.

Although our results indicated the capability of ^68^Ga-5 in the detection of PFKFB3-positive tumors, relatively higher background uptake was observed in both biodistribution and PET imaging studies, which may be caused by the lipophilicity of this tracer. With a *log D*_*7.4*_ value of −1.6 for ^68^Ga-5, significant liver uptake was detected, as well as higher intestine and urinary system distribution, and further investigation and optimization should pay more attention to the physicochemical properties to accelerate the excretion of the desired compound to reduce the background uptake, and enhance the “Tumor-to-Background” ratios. Furthermore, with DOTA as a chelator for radio-metal labeling, therapeutic radionuclides can also be radiolabeled for tumor treatment *via* this scaffold. Table 1.

**TABLE 1:** The IC_50_ of compounds 4 and 5 in biochemical activity and cellular inhibition assays.

Compound	IC_50_ for PFKFB3 activity inhibition (nM)*	IC_50_ for F2,6BP production (µM)*
4	6.7 ± 2.3	2.3 ± 0.8
5	12.5 ± 4.5	5.3 ± 1.4

*All tests were performed in triplicate.

## 5 Conclusion

In conclusion, we have successfully prepared the ^68^Ga-labeled aminoquinoxaline derivative (^68^Ga-5) as a potential PFKFB3 PET tracer, and our preliminary evaluations indicated that although this scaffold showed potent activity against PFKFB3 *in vitro* and *in vivo*, further investigation and optimization, focused on the physicochemical parameters of this scaffold, would be needed to develop more successful PFKFB3 targeted PET tracer for tumor diagnosis and treatment.

## Data Availability

The datasets presented in this study can be found in online repositories. The names of the repository/repositories and accession number(s) can be found in the article/Supplementary Material.
