# Supplementary material for: Synthesis, radiolabeling, and evaluation of 68Ga-labeled aminoquinoxaline derivative as a potent PFKFB3-targeted PET tracer
Source: Front Chem. 2023 Mar 22;11:1158503. doi: 10.3389/fchem.2023.1158503 (PMC10073729; doi:10.3389/fchem.2023.1158503)
Supplement: Supplementary file 1 [file DataSheet1.docx]

*Supplementary Materials for* RadioSynthesis and preliminary evaluations of 68Ga-labeled aminoquinoxaline derivative as a potential PFKFB3 PET tracer.

Feng Chen^1, 2, 3, 4^, Yi Wu^3#^, Honghai Yin^5^, Feijing Su^6^, Rui Huang^7^, Xiaoai Wu^5^ and Qian Liu^1, 4, 8, 9#^

Table of contents:

[Scheme S1. Chemical synthesis route for compound 5. 3](#_Toc125192390)

[Synthesis and Characterization of compound 7. 4](#_Toc125192391)

[Synthesis and Characterization of compound 8. 5](#_Toc125192392)

[Synthesis and Characterization of compound 9. 6](#_Toc125192393)

[Synthesis and Characterization of compound 11. 7](#_Toc125192394)

[Synthesis and Characterization of compound 12. 8](#_Toc125192395)

[Synthesis and Characterization of compound 13. 9](#_Toc125192396)

[Synthesis and Characterization of compound 14. 10](#_Toc125192397)

[Synthesis and Characterization of compound 5. 11](#_Toc125192398)

Scheme S1. Chemical synthesis route for compound 5.

**Regents and conditions:** a. iron powder, H_2_O, EtOH, 78°C, 3 h; b. 1,4-dioxane-2,3-diol, EtOH, r.t., overnight; c. Pd(dppf)Cl_2_, DIPEA, 1,4-Dioxane, H_2_O, N_2_, 85°C, 4 h; d. EDCI, HOBT, 4-Methylmorpholine, tert-butyl 3-aminopiperidine-1-carboxylate, r.t, 4 h; e. iron powder, H_2_O, EtOH, 78°C, 4 h; f. tBuOK, Pd(OAc)_2_, BINAP, toluene, 100°C, overnight; g. TFA, CH_2_Cl_2_, r.t, overnight; h. DOTA-GA anhydride, Et_3_N, DMSO, r.t, overnight.

Synthesis and Characterization of compound 7.

*3,5-dibromobenzene-1,2-diamine (7).* To a suspension of 2,4-dibromo-6-nitroaniline (4.0 g, 13.5 mmol) and iron powder(4.5 g, 80.6 mmol) in a 3:1 mixture of EtOH and H2O was added ammonium chloride (723 mg, 13.5mol). The reaction mixture was kept sitrring for 4 hours under 78°C. Filtration of the mixture through a diatomite plug to remove the iron powder, and evaporation of the solvents afforded crude product, which was purified on a silica gel column with 10-15% EtOAc in hexanes to afford compound 7 as a yellow solid (2.8 g, yield 78.2%) ^1^H NMR (400 MHz, Chloroform-*d*) δ 7.10 (d, *J* = 2.1 Hz, 1H), 6.78 (d, *J* = 2.1 Hz, 1H), 3.64 (s, 4H).


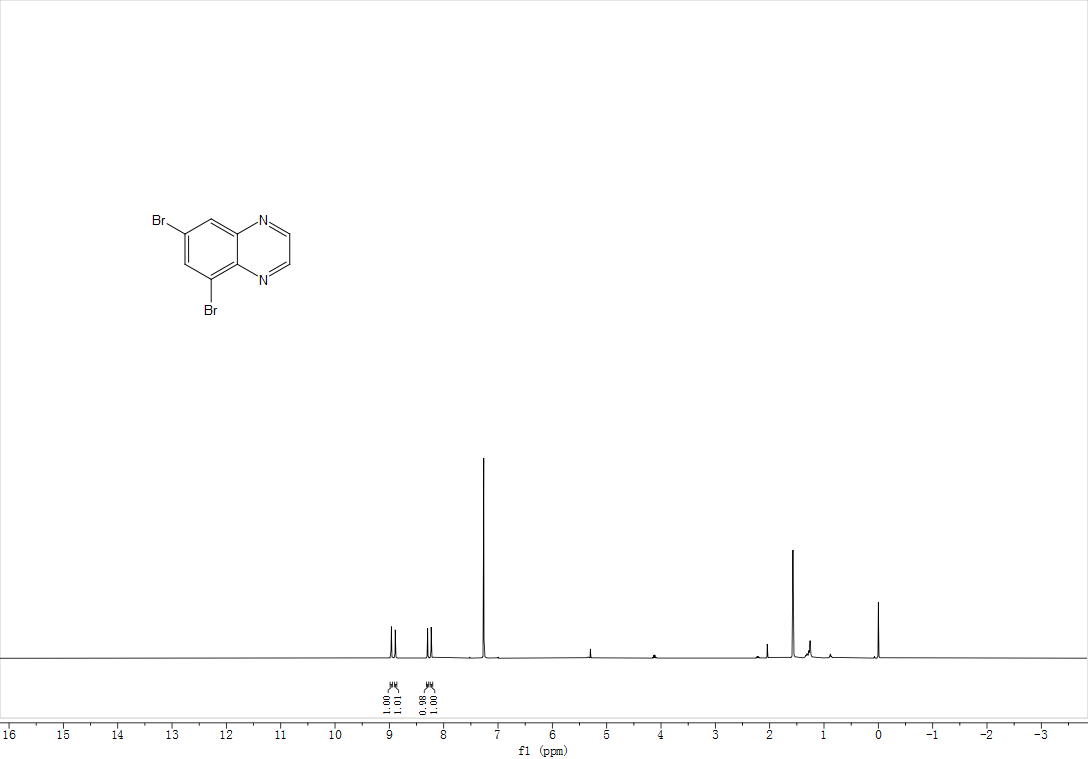


Synthesis and Characterization of compound 8.

*5,7-dibromoquinoxaline (****8****).* To a solution of compound **7** (2.8 g, 10.5mmol) in 20 mL of EtOH was added 1,4-dioxane-2,3-diol (1.9 g, 15.7mmol), and the mixture was kept stirring at roomtemperature overnight. TLC indicates the conversion was complete and filtration of the reaction mixture to get the white solid. Washed with EtOH (2 * 10), the solid was dryed under reduced pressure to get the compound 8 as final product which was used in the next step without further purification (1.8 g, yield 60.1%). ^1^H NMR (400 MHz, Chloroform-*d*) δ 8.97 (d, *J* = 1.8 Hz, 1H), 8.89 (d, *J* = 1.8 Hz, 1H), 8.30 (d, *J* = 2.1 Hz, 1H), 8.23 (d, *J* = 2.1 Hz, 1H).


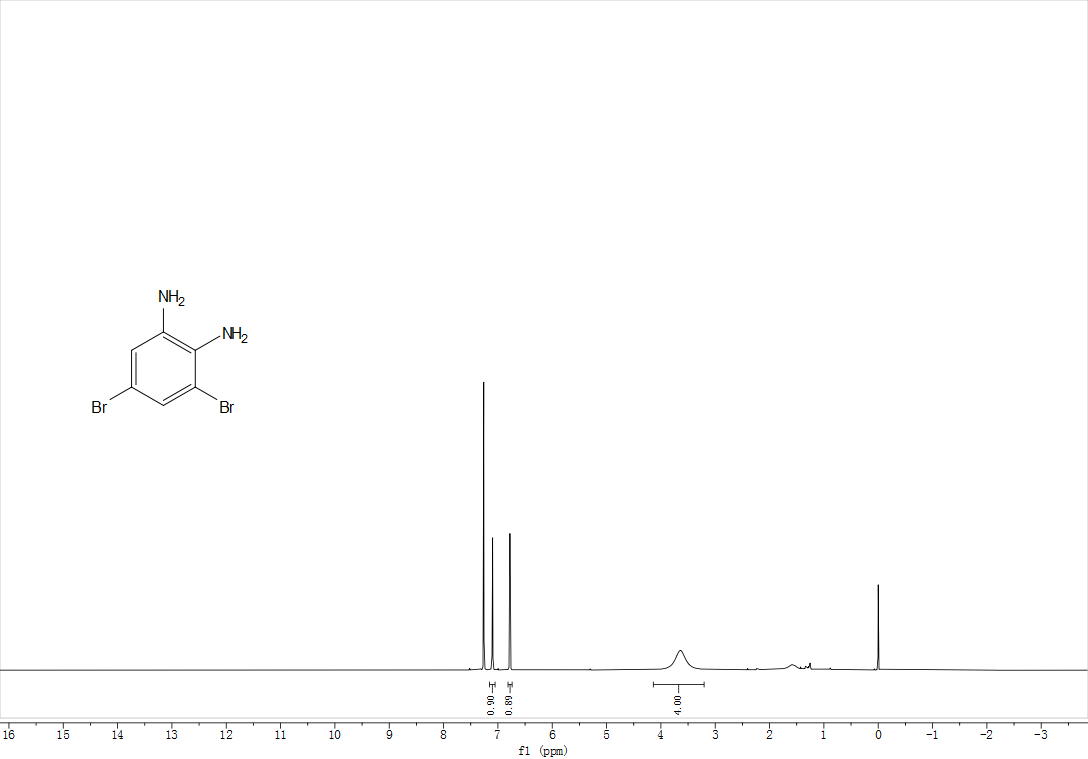


Synthesis and Characterization of compound 9.

*7-bromo-5-(1-methyl-1H-indol-6-yl)quinoxaline (9).* Compound 8 (1.8 g, 6.2 mmol), 1-methyl-6-(4,4,5,5-tetramethyl-1,3,2-dioxaborolan-2-yl)-1H-indole (1.3 g, 2.0 mmol), Pd(dppf)Cl2 (456 mg, 0.6 mmol), DIPEA (2.1 mL, 12.5 mmol) were dissolved in 20 mL of 1,4-dioxane and 20 mL of H2O under nitrogen and stirring at 85 °C overnight. After 4 hours, the reaction mixture was added water (15 ml) and extracted with EtOAc (25 ml * 2). The organic extracts were combined and washed with saturated sodium chloride solution, dried over Na2SO4 and concentrated in vacuo. The crude product was then purified on a silica gel column with 10-20% EtOAc in hexane to afford compound 9 as a yellow solid (697 mg, yield 33.0%). ^1^H NMR (400 MHz, Chloroform-*d*) δ 8.92 (dd, *J* = 13.6, 1.8 Hz, 2H), 8.52 (d, *J* = 2.0 Hz, 1H), 8.37 (d, *J* = 2.0 Hz, 1H), 7.76 (d, *J* = 8.3 Hz, 1H), 7.69 (s, 1H), 7.52 (dd, *J* = 8.2, 1.6 Hz, 1H), 7.16 (d, *J* = 3.1 Hz, 1H), 6.55 (d, *J* = 3.3 Hz, 1H), 3.90 (s, 3H).

^
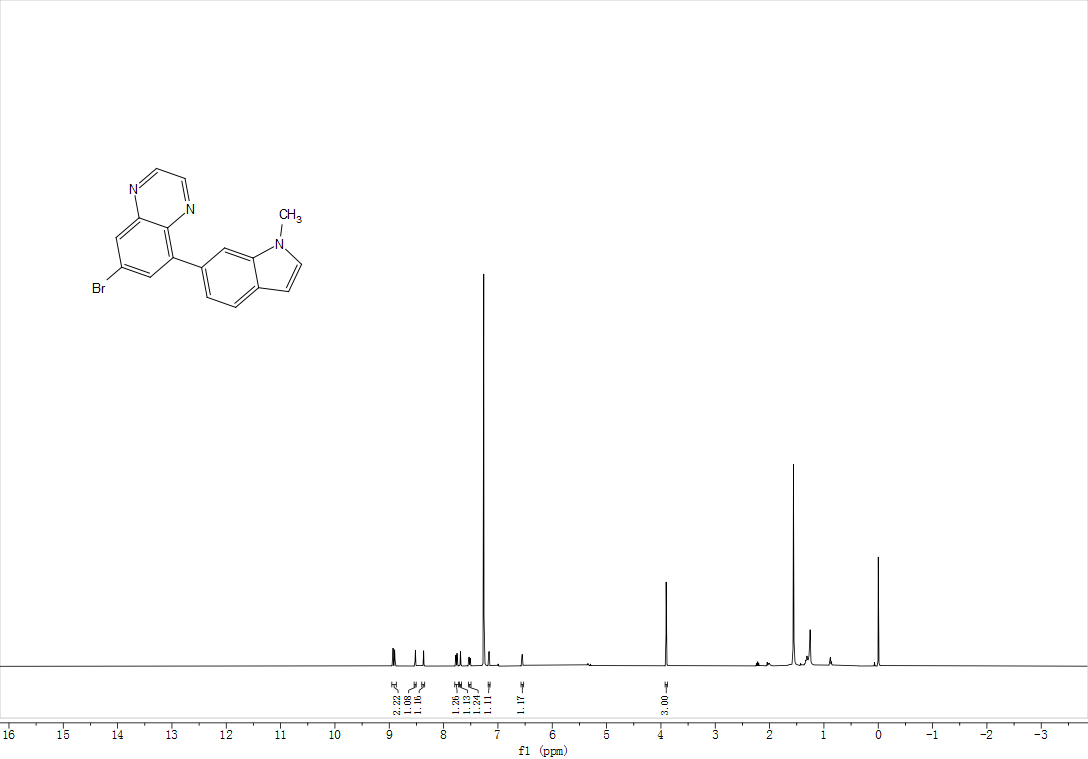
^

Synthesis and Characterization of compound 11.

*Tert-butyl 3-(3-nitrobenzamido)piperidine-1-carboxylate (11).* 5-nitronicotinic acid (1 g, 5.9 mmol), EDCI (1.37g，7.1mmol) and HOBT (964 mg, 7.1 mmol) were dissolved in 20 mL of DMF and kept stirring at roomtemperature for 1 hour. N-Methylmorpholine (1.5 ml, 14.3 mmol) and tert-butyl 3-aminopiperidine-1-carboxylate (1.2g, 7.1mmol) were added into the reaction mixture, and the reaction mixture was kept stirring for an addition 4 hours. The reaction mixture was added water (30 ml) and extracted with EtOAc (30 ml * 2). The organic extracts were combined and washed with saturated sodium chloride solution, dried over Na2SO4 and concentrated in vacuo. The crude product was then purified on a silica gel column with 1-5% MeOH in DCM to afford compound 11 as a yellow solid (1.82 g, yield 87.6%). ^1^H NMR (400 MHz, DMSO-*d*_6_) δ 9.49 (d, *J* = 2.5 Hz, 1H), 9.42 (d, *J* = 1.9 Hz, 1H), 9.09 (d, *J* = 7.8 Hz, 1H), 9.00 (s, 1H), 3.90 – 3.70 (m, 2H), 2.92 – 2.72 (m, 2H), 1.96 – 1.87 (m, 1H), 1.81 – 1.72 (m, 1H), 1.66 – 1.54 (m, 1H), 1.39 (s, 9H), 1.23 (s, 2H).


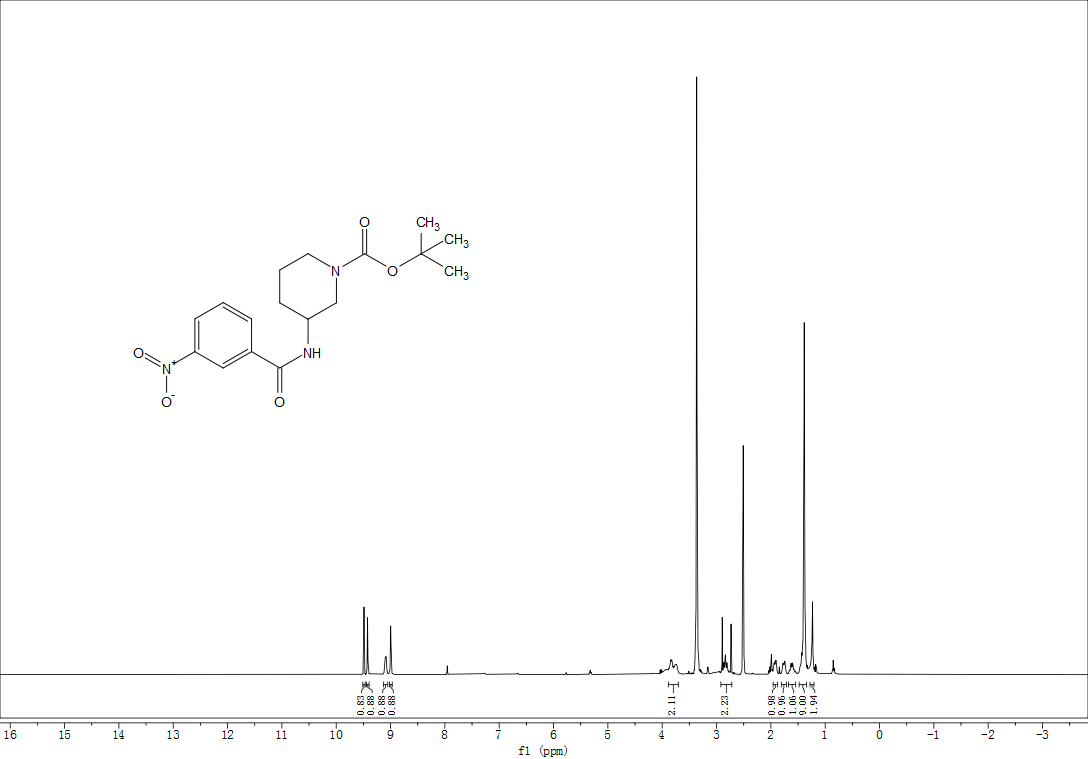


Synthesis and Characterization of compound 12.

*Tert-butyl 3-(3-aminobenzamido)piperidine-1-carboxylate (12).* To a suspension of compound 11 (1.82 g, 5.2 mmol) and iron powder(1.7 g, 31.3 mmol) in a 3:1 mixture of EtOH and H2O (40 mL) was added ammonium chloride (278 mg, 5.2 mmol). The reaction mixture was kept sitrring for 4 hours under 78°C. Filtration of the mixture through a diatomite plug to remove the iron powder, and evaporation of the solvents afforded crude product, which was purified on a silica gel column with 1-10% MeOH in DCM to afford compound 12 as a yellow solid (1.49 g, yield 89.6%) ^1^H NMR (400 MHz, Chloroform-*d*) δ 7.10 (d, *J* = 2.1 Hz, 1H), 6.78 (d, *J* = 2.1 Hz, 1H), 3.64 (s, 4H). ^1^H NMR (400 MHz, DMSO-*d*_6_) δ 8.26 (d, *J* = 7.4 Hz, 1H), 8.15 (d, *J* = 1.9 Hz, 1H), 8.02 (d, *J* = 2.7 Hz, 1H), 7.27 (s, 1H), 5.49 (s, 2H), 3.74 (d, *J* = 9.0 Hz, 2H), 3.17 (d, *J* = 5.1 Hz, 1H), 3.01 – 2.56 (m, 2H), 1.94 – 1.80 (m, 1H), 1.72 (d, *J* = 12.6 Hz, 1H), 1.61 – 1.45 (m, 1H), 1.38 (s, 9H), 1.24 (s, 1H).

^
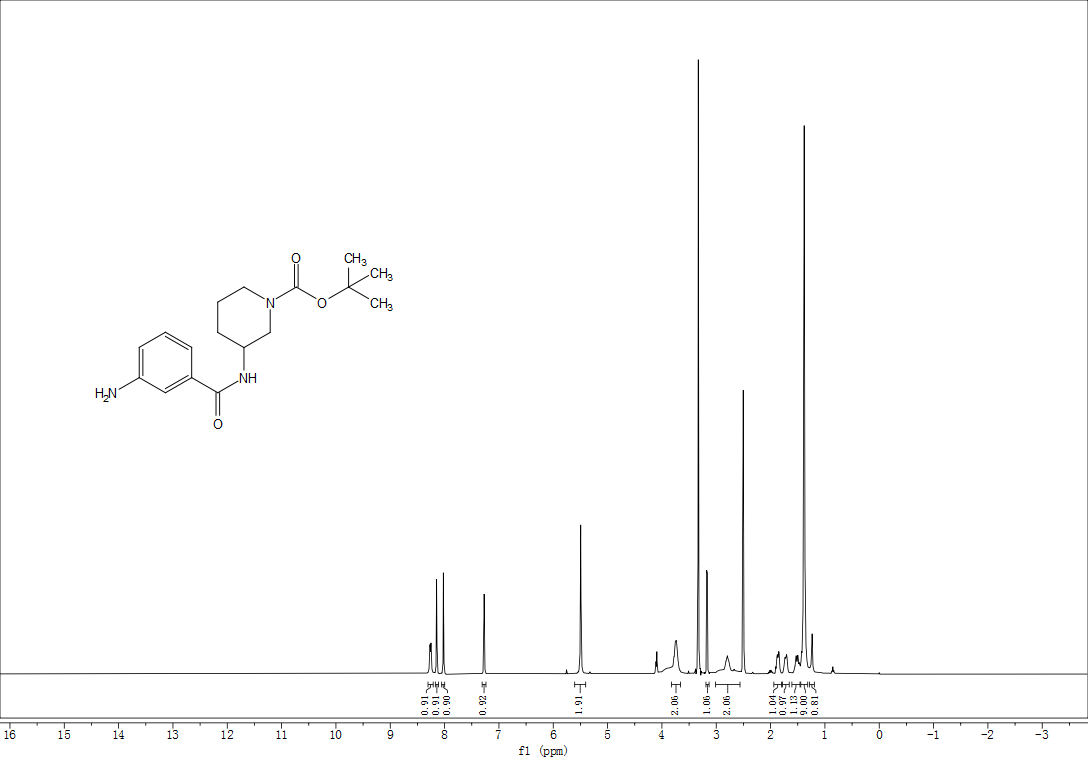
^

Synthesis and Characterization of compound 13.

*Tert-butyl 3-(5-((8-(1-methyl-1H-indol-6-yl)quinoxalin-6-yl)amino)nicotinamido)piperidine-1-carboxylate (13).* Compound 9 (697 mg, 2.1 mmol), compound 12 (661 mg, 2.1 mmol), potassium tert-butoxide (371 mg 3.3mmol), Pd(OAc)2 (46 mg,0.21mmol) and BINAP (129 mg, 0.21 mmol) were dissolved in toluene (15 ml) under nitrogen and stirring at 100 °C overnight. Toluene was removed under reduced pressure, and the crude product was purified on a silica gel column with 1-5% MeOH in DCM to afford compound 13 as a yellow solid (690 mg, yield 57.8%). ^1^H NMR (400 MHz, Chloroform-*d*) δ 8.91 (d, *J* = 1.9 Hz, 1H), 8.89 (d, *J* = 2.7 Hz, 1H), 8.68 (d, *J* = 1.9 Hz, 1H), 8.58 (d, *J* = 1.9 Hz, 1H), 8.25 (t, *J* = 2.3 Hz, 1H), 8.19 (s, 1H), 7.95 – 7.88 (m, 2H), 7.72 (d, *J* = 8.2 Hz, 1H), 7.70 – 7.66 (m, 1H), 7.48 (dd, *J* = 8.2, 1.6 Hz, 1H), 7.13 (d, *J* = 3.1 Hz, 1H), 6.53 (dd, *J* = 3.1, 0.8 Hz, 1H), 5.30 (s, 1H), 3.89 (s, 3H), 3.41 (d, *J* = 115.4 Hz, 4H), 1.88 (s, 1H), 1.69 (s, 4H), 1.60 (s, 1H), 1.45 (s, 9H).


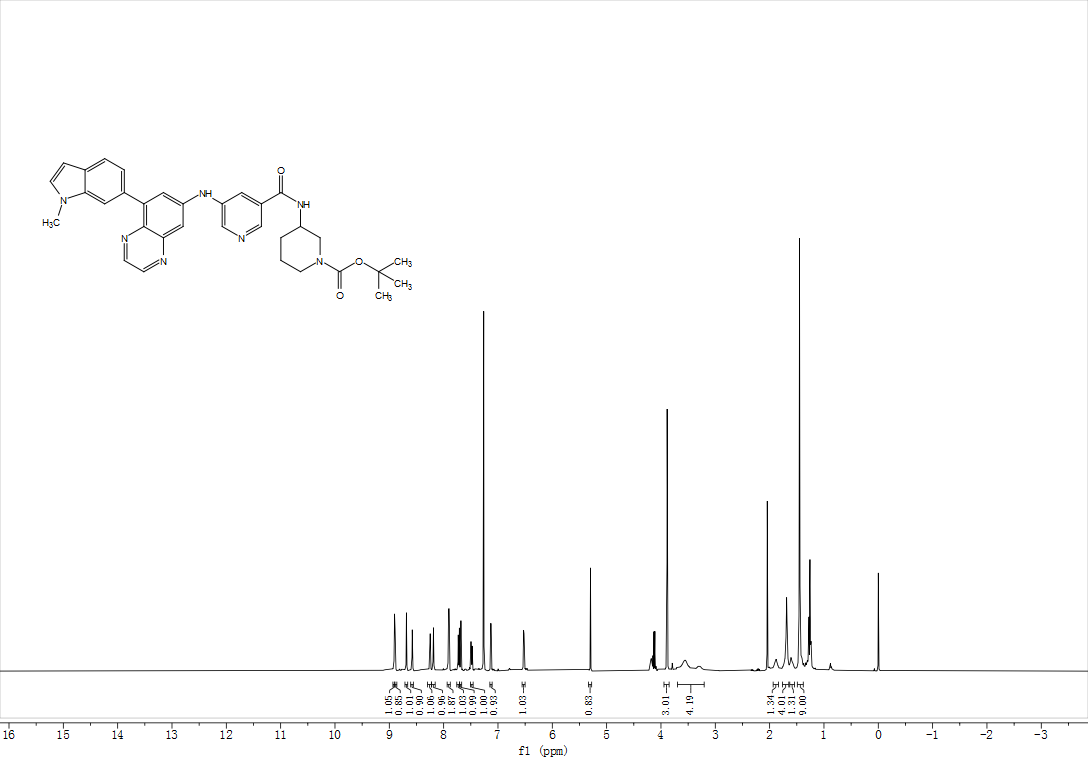


Synthesis and Characterization of compound 14.

*5-((8-(1-methyl-1H-indol-6-yl)quinoxalin-6-yl)amino)-N-(piperidin-3-yl)nicotinamide (14).* Compound 13 (690 mg, 1.2 mmol) was dissolved in DCM (10 mL), and the mixture was added 1.8 mL of TFA (24.7 mmol) dropwise and stirring overnight at roomtemperature. The organic solvent was removed under reduced pressure and the crude product was purified on a silica gel column with 10-15% MeOH in DCM to afford compound 14 as a yellow solid (368 mg, yield 64.6%). ^1^H NMR (400 MHz, DMSO-*d*_6_) δ 9.27 (s, 1H), 9.06 – 9.00 (m, 2H), 8.89 (dd, *J* = 3.6, 2.3 Hz, 3H), 8.75 – 8.64 (m, 2H), 8.37 (t, *J* = 2.2 Hz, 1H), 7.91 (d, *J* = 2.1 Hz, 3H), 7.68 (d, *J* = 8.3 Hz, 1H), 7.50 – 7.40 (m, 2H), 6.49 (dd, *J* = 3.1, 0.9 Hz, 1H), 4.24 – 4.15 (m, 1H), 3.90 (s, 3H), 3.37 (dd, *J* = 12.3, 3.8 Hz, 1H), 3.22 (d, *J* = 12.9 Hz, 2H), 2.87 (q, *J* = 10.5 Hz, 3H), 1.93 (tt, *J* = 14.7, 3.4 Hz, 2H).


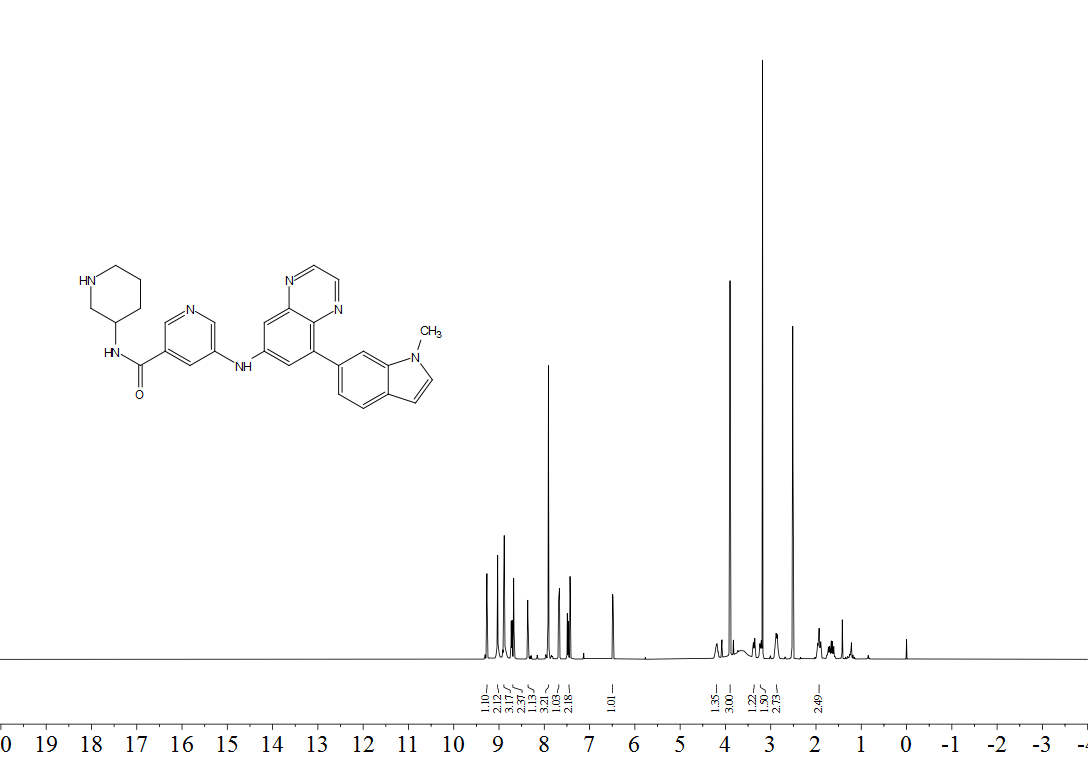


Synthesis and Characterization of compound 5.

*2,2',2''-(10-(1-carboxy-4-(3-(5-((8-(1-methyl-1H-indol-6-yl)quinoxalin-6-yl)amino)nicotinamido)piperidin-1-yl)-4-oxobutyl)-1,4,7,10-tetraazacyclododecane-1,4,7-triyl)triacetic acid (5).* Compound 14 (30 mg, 0.06 mmol), DOTA-GA anhydride (32 mg，0.07 mmol) was dissovled in 1 mL of DMSO, and Et3N (10 µL，0.07 mmol) was added into the mixture. The reaction mixture was kept stirring overnight at roomtemerature. The product was purified on an Agilent 1100 system equiped with a C-18 column (Waters SunFire Prep C18 5µm, 250*10 mm) and eluted with 10-90% 0.1% HCOOH in water and 90-10% MeCN from 0-20 min, at a flowrate of 4 mL/min (retention time for product was 10.0-10.5 min). The final product (32.5 mg, yield 55.3%) was obtained via freeze drying from the HPLC fraction. ^1^H NMR (400 MHz, DMSO-d6) δ 9.20 (t, J = 4.9 Hz, 1H), 9.01 (d, J = 2.0 Hz, 1H), 8.87 (d, J = 2.3 Hz, 2H), 8.68 – 8.61 (m, 1H), 8.38 (d, J = 16.0 Hz, 1H), 7.95 – 7.83 (m, 3H), 7.67 (d, J = 8.3 Hz, 1H), 7.52 – 7.39 (m, 2H), 6.48 (d, J = 3.1 Hz, 1H), 4.48 – 2.53 (m, 30H), 1.97 – 1.00 (m, 9H). ^13^C NMR (101 MHz, DMSO-*d*_6_) δ144.91, 144.91, 144.91, 142.13, 142.13, 142.13, 140.59, 140.59, 140.59, 138.13, 136.97, 136.97, 136.97, 136.97, 135.27, 135.27, 135.27, 135.27, 131.33, 131.33, 130.89, 130.89, 129.48, 126.54, 126.54, 126.54, 126.54, 119.23, 119.23, 119.23, 119.23, 116.91, 116.91, 116.91, 116.91, 108.52, 108.52, 107.11, 107.11, 107.11, 107.11, 98.67, 98.67, 98.67, 30.93(4C). MS(ESI): m/z calculated for [C_47_H_58_N_11_O_10_]^+^: 935.42 [M+H]^+^; found: 935.9, HRMS: m/z calculated for [C_47_H_55_N_11_O_10_K]^-^: 972.3770 [M-2H+K]; found: 972.3691.

^
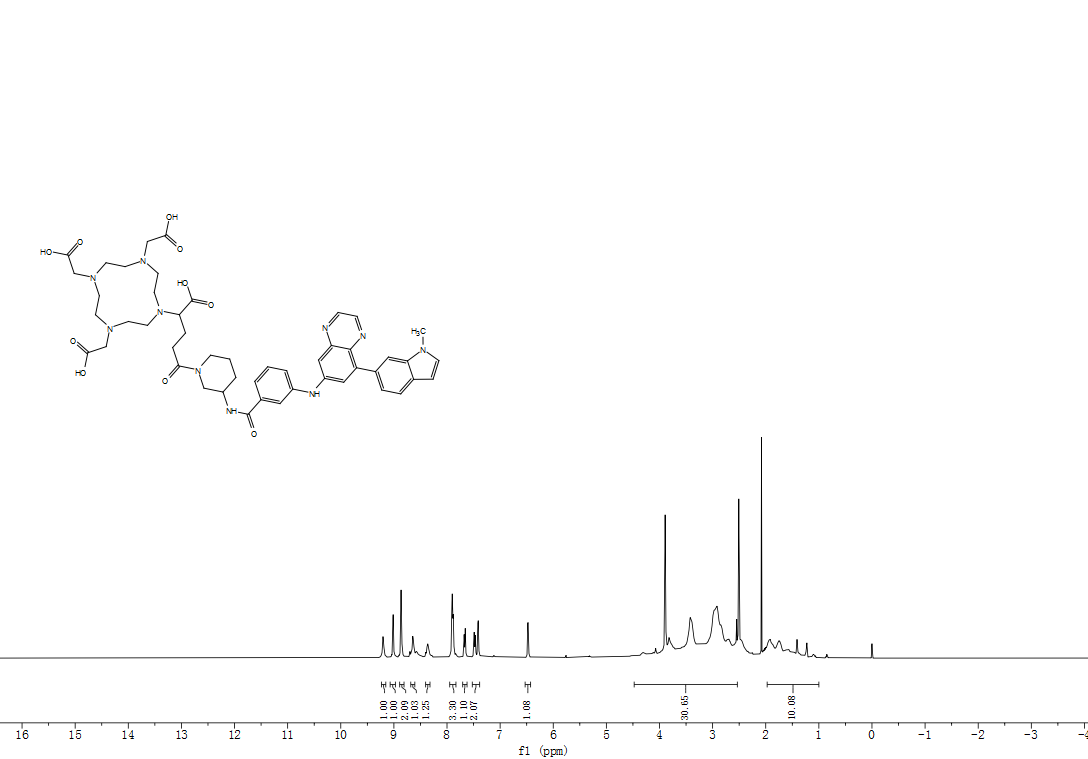
^

^
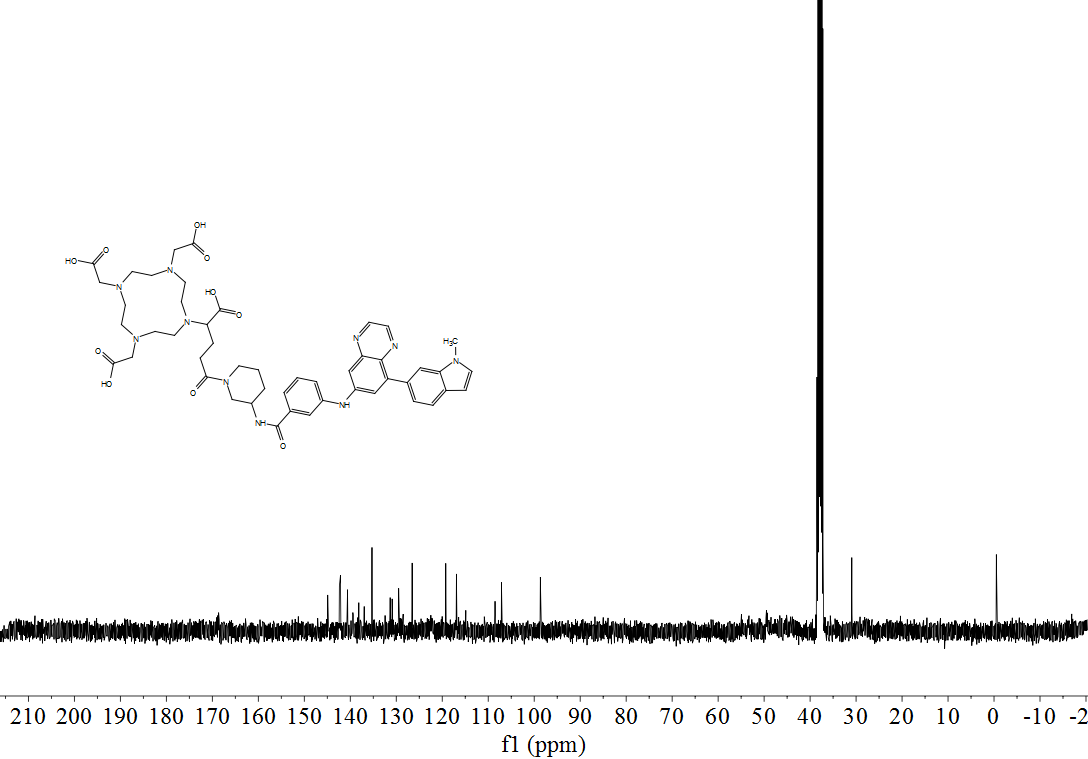
^

**^ESI-MS^**


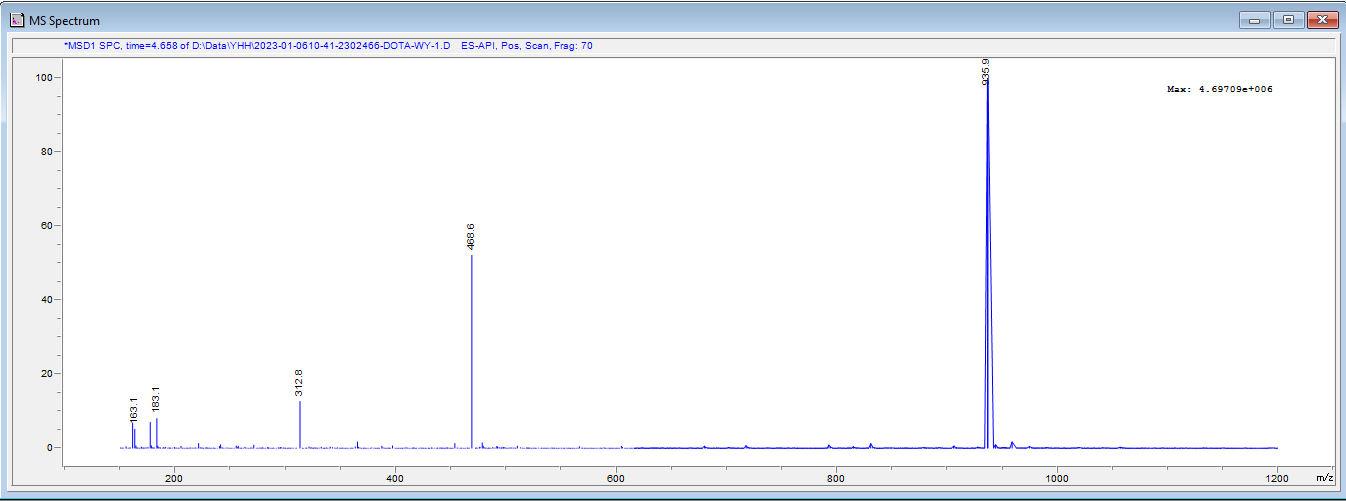


HRMS
